# Real-time beam shaping without additional optical elements

**DOI:** 10.1038/s41377-018-0014-0

**Published:** 2018-06-20

**Authors:** Felix Fries, Markus Fröbel, Pen Yiao Ang, Simone Lenk, Sebastian Reineke

**Affiliations:** Dresden Integrated Center for Applied Physics and Photonic Materials (IAPP), Technische Universität Dresden, Nöthnitzer Straße 61, Dresden, 01187 Germany

## Abstract

Providing artificial light and enhancing the quality of the respective light sources is of continued interest in the fields of solid state, condensed matter, and semiconductor physics. Much research has been carried out to increase the luminous efficiency, lifetime and colour stability of such devices. However, the emission characteristics of a given light source do not necessarily comply with today’s often sophisticated applications. Here, beam shaping addresses the transformation of a given light distribution into a customized form. This is typically achieved by secondary optical elements often sporting elaborate designs, where the actual light source takes up only a small fraction of the system’s volume. Such designs limit the final light source to a single permanent operation mode, which can only be overcome by employing mechanically adjustable optical elements. Here we show that organic light-emitting diodes (OLEDs) can enable real-time regulation of a beam shape without relying on secondary optical elements and without using any mechanical adjustment. For a red light-emitting two-unit OLED architecture, we demonstrate the ability to continuously tune between strongly forward and strongly sideward emission, where the device efficiency is maintained at an application-relevant level ranging between 6 and 8% of external quantum efficiency for any chosen setting. In combination with additional optical elements, customizable and tuneable systems are possible, whereby the tuning stems from the light source itself rather than from the use of secondary optics.

## Organic Light-Emitting Diodes: Promising beam-shaping properties uncovered

A simple, efficient design incorporating organic light-emitting diodes could enable the development of customized, adjustable light sources for many applications. The ability to manipulate the shape of a beam of light emitted by a device is useful in many scenarios, for example switching between normal and private screen display modes on a mobile phone. However, current beam-shaping designs require extra components, making devices bulkier and more complex. Now, Felix Fries, Sebastian Reineke and co-workers at the Technische Universität Dresden, Germany, have used organic light-emitting diodes (OLEDs) to generate real-time beam shaping simply by manipulating the input power signal. The team’s design comprises two red-emitting OLEDs; by sending a carefully-modulated power signal through both OLEDs simultaneously they could customize the resulting beam of light. They hope to expand their research into full red-green-blue colour displays.

## Introduction

Beam shaping is a prominent topic in various fields of optics. In laser physics, optical elements are commonly used to transform a Gaussian-shaped TEM00 mode into various spatial distributions that are defined by the application requirements^1^. For such developments, sophisticated theoretical analysis and elaborate designs of phase plates are utilized^2^. Inorganic LEDs are point-sources in most settings, and are therefore easily and effectively combined with secondary beam-shaping systems^3^, metallic nanostructures or optical nanoantennas^4^. OLEDs, being area light sources with broad-band emission^5^ and often Lambertian emission characteristics, have not been subject to serious beam-shaping designs. Rather, efforts in developing elaborate optical concepts aim to increase the outcoupling efficiency, which for planar devices, ranges only between 20 and 30%^5,6^. A few studies focus on OLED-based beam shaping by moving away from the planar architecture^7^, employing diffractive gratings to achieve a very directional emission^8^, or by reducing the size of OLED pixels such that, when combined with microlens arrays, their output resembles that of a point source^9^. What all of the above concepts have in common is the fact that only one final light distribution is realized, which is defined by the optical interplay between light source and secondary optical elements. To circumvent this limitation, mechanically tuneable elements are necessary, which result in a setup that is much more complicated^1^. While some applications can easily integrate elaborate optical designs with movable parts, our concept can be used in scenarios that cannot tolerate bulky components. OLEDs are currently entering the market as automotive rear lights, where the additional beam shaping would allow for light tracking of the road and the following car.

When used as a display pixel, the beam-shaping OLED design can allow for continuous switching between normal and privacy screen mode by controlling the angular emission pattern. The fast switching of the emission pattern of a light source can further be utilized in rapid surface meteorology, e.g., in quality control applications. Here, simple and cost-effective solutions with a small system footprint are necessary.

We have revisited the possibilities of OLED technology and have, through careful optical design, developed an OLED-based area light source with high efficiency, good colour stability and most importantly, significant angular tunability for the emitted light. Neither passive optical elements nor movable parts are needed and the beam shape can be altered continuously during operation. This is why we refer to our concept as active beam shaping.

## Materials and methods

All simulations were carried out using simulation software developed in-house. Theoretical details can be found in the literature^10,11^. The simulation solves the Maxwell equation for stratified media, assuming that the emission layer is infinitesimally thin; thus, the system of equations is solved using Green functions^12^. Parameters that have to be set, are the thickness, the refractive index *n*(*λ*), and the absorption coefficient *k*(*λ*) of each layer. Furthermore, both the PL-spectrum and the molecular orientation of the emitter must be known. The software itself has proven functionality with various types of planar devices^10,13^ and even works for corrugated samples^14^.

All samples are produced on glass substrates pre-coated with ITO anodes. Organic layers and metals are deposited by thermal evaporation in a UHV chamber at a base pressure of 10^−6^–10^−7^ mbar and rates of 0.2–2 Å/s. The samples are encapsulated to prevent exposure to oxygen, water, and dust. The OLEDs follow the pin-architecture. Two stacked OLEDs are evaporated directly on top of each other, separated by a Au/Ag wetting layer^15^. The hole transport layers (HTLs) consist of 4 weight percent (wt%) 2,2′-(perfluoronaphthalene-2,6-diylidene)dimalononitrile (F_6_-TCNNQ) doped into 2,2′,7,7′-tetrakis-(N,N-dimethylphenylamino)−9,9′-spirobifluoren (Spiro-TTB). Electron transport layers (ETLs) consist of caesium-doped 4,7-diphenyl-1,10-phenanthroline (BPhen). Regarding energy barriers^16^ and chemical interactions^17^, the blocking layer materials are chosen from the following pool: BPhen or aluminium(III)bis(2-methyl-8-quninolinato)−4-phenylphenolate (BAlq2) as the hole blocking layer (HBL) and 2,2′,7,7′-tetrakis-(N,N-diphenylamino)−9,9′-spirobifluorene (Spiro-TAD) or N,N’-di(naphthalen-1-yl)-N,N’-diphenyl-benzidine (NPB) for the electron blocking layer (EBL). Red emission is obtained by using either iridium(III)bis(2-methyldibenzo-[f,h]quinoxaline)(acetylacetonate) (Ir(MDQ)_2_(acac)) or tris(1-phenylisoquinoline)iridium(III) (Ir(piq)_3_). Both are phosphorescent emitters, which are doped into a matrix of NPB, each at a concentration of 10 wt%. Details of the device architecture and layer thicknesses are provided in the supplementary information, Fig. S1.

Even though the final device is AC driven to enable active beam shaping, a complete characterization of each subunit is carried out under DC conditions. Electro-optical performance, as current and luminance against voltage, is measured using a source measure unit (SMU2400, Keithley Instruments, Cleveland, Ohio, USA) combined with a calibrated spectrometer (CAS140, Instrument Systems GmbH, Munich, Germany). Angularly resolved measurements are carried out using a spectro-goniometer setup that measures the irradiance with a calibrated USB-spectrometer (USB4000, Ocean Optics, Inc., Dunedin, Florida, USA). Here, the OLED is mounted onto a rotary table, and the spectrometer is placed at a constant distance from the device. The information from this measurement is also used to calculate correct efficiency values, which are, in this case, indispensable due to the highly non-Lambertian behaviour of the samples. To obtain an image of the beam shape, another setup was developed. In particular, the OLED illuminates a flat, diffusive screen. Using a digital single-lens reflex camera (EOS-D30, Canon, Krefeld, Germany) and subsequent image evaluation tools, the brightness distribution can be analyzed. For evaluation, the spectra are flattened using an FFT low-pass filter. Asymmetries in the profiles suggest that the screen is not perfectly parallel to the OLED. Therefore, the mean value of both maxima has to be used.

The power supply for the PWM consists of an arbitrary waveform generator (33220A, Agilent Technologies, Santa Clara, California, USA) and a subsequent 5 MHz high-voltage amplifier (WMA 300, Falco Systems, Amsterdam, The Netherlands).

## Results and discussion

**Fig. 1:**
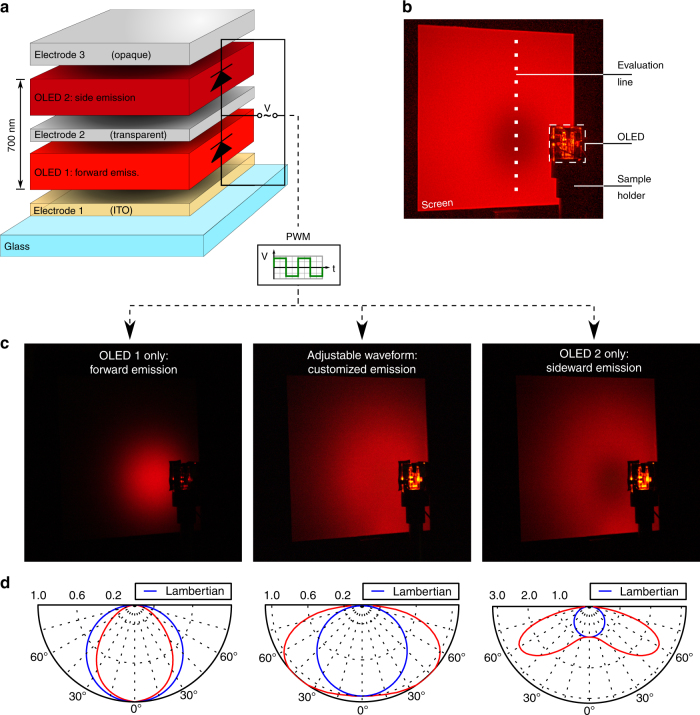
Working scheme for active beam-shaping OLEDs. **a** Two stacked OLED units, electrically connected for AC operation; **b** Experimental setup for analysis of the brightness distribution on a planar, two-dimensional surface. The OLED and the screen have to be placed parallel to each other. The evaluation of the brightness is done in the vertical direction, as indicated by the dotted line; **c** Using PWM, different AC signals can be applied to the device, resulting in either a ring-shaped beam (left), broad homogeneous illumination (centre) or a focused spot (right); **d** Respective irradiance as a function of the viewing angle for the conditions shown in **c**, measured using a spectro-goniometer

Figure 1a shows the general device layout used in this study (for details of the OLED architecture, refer to Fig. S1 in the Supplementary Information). Two subunits are stacked vertically^18,19^ in a configuration that allows for emission through the substrate, i.e., a bottom emitting layout. Compared to conventional OLEDs, this device shows an increased overall thickness of ~800 nm, which is needed to realize the desired optical effects. Here, electrically doped charge transport layers enable free design of the optical system without sacrificing electrical performance^10^. For this beam-shaping concept to work, it is important to be able to address each of the units independently. In this study, the tandem device utilizes an AC/DC driving concept^20^, where the outermost electrodes sit on a common potential. Hereby, this two-unit device can be operated with only two electrodes, making use of pulse width modulation (PWM) to control the emission ratio of OLED 1 and OLED 2. This device concept can be equally implemented using the conventional vertical design sporting three independent electrodes^18,19^, as the electrode configuration has no influence on the optical effects at play. For visualization of the tuneable emission, a setup is chosen in which the OLED emission is projected onto a planar white screen, as illustrated in Fig. 1b. Three different driving scenarios are illustrated in Fig. 1c: Addressing only OLED 1, a central spot is observed on the projection screen. In contrast, the emission pattern changes to a distinct ring shape in the case of addressing only OLED 2. Finally, by combining the time-averaged emission from OLED 1 and OLED 2 via PWM, a customized emission pattern can be realized. The switching ratio in such a device is only limited by the time needed to reach a steady state in the actively emitting unit, which is in the 5 µs range for phosphorescent OLEDs^21^ and even nanoseconds for fluorescent OLEDs^22^. High-speed beam-shaping solutions in the literature demonstrate switching velocities in the same regime or below (kHz - MHz)^23–25^. Characterization of the emission profile for the unit is carried out using a spectro-goniometer, where a detector measures the angular-dependent emission intensity. The results are shown in Fig. 1d. The sideward-emitting OLED 2 shows an emission intensity maximum at 56°, showing a 2.7-fold increase compared to the emission at 0°. At this angle, the forward-emitting unit provides only 30% of its own intensity at 0°, indicating a very clear separation of the two extreme operation modes. A more detailed stepping of the PWM-controlled angular emission profiles is summarized in the Supplementary Information, Fig. S2 and S3. Additionally, an animated graphics interchange format (GIF) file summarizing the projections observed on the screen for a sweep of different driving conditions is available in the Supplementary Material.

**Fig. 2:**
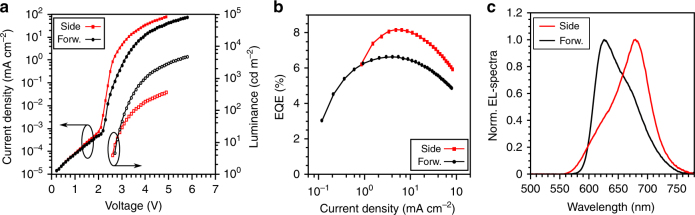
Electro-optical device characteristics. **a** The current density as a function of the driving voltage is shown in black, with the respective forward luminance in red; **b** The resulting EQE for both units; **c** EL-spectra for both the side and the forward emission unit at their emission maximum. The peaks show a spectral distance of 54 nm

Figure 2 contains the key performance characteristics for the red, active beam-shaping OLEDs. Figure 2a shows current density (*j*)-voltage (*V*)-forward luminance characteristics for both units independently. Very steep *j-V* characteristics are observed despite the very large overall device thickness (~800 nm), which is made possible by the use of highly conductive transport layers^26^. The forward luminance for OLED 2 is, of course, lower as it is designed to emit efficiently only at high angles of observation. The external quantum efficiency (EQE) versus *j* is shown for both units independently in Fig. 2b, with a maximum value of 6.6 and 8.2% for the forward-emitting OLED 1 and the sideward-emitting OLED 2, respectively. The EQE of the forward-emitting unit is limited here, because the photoluminescence quantum yield for the emitter used (Ir(piq)_3_) is only ~50%^27,28^. Figure 2c shows the electroluminescence (EL) spectra for OLED 1 and OLED 2 at their respective maximum intensity emission, which are spaced apart by 54 nm.

The final OLED stack for effective beam shaping should show very anisotropic emission patterns for the two units employed. Therefore, one needs to analyze the explicit expression for the spectral radiant intensity (SRI) per unit area *I*(*λ*, *θ*), as given by^10^:

1 $$I\left( {\lambda ,\theta } \right) = \frac{hc}{\lambda }\frac{I}{e}\gamma s_{\rm{EL}}\left( \lambda \right)\underbrace {\eta _{{\rm rad}}^ \ast \left( \lambda \right)\frac{{P_{{\rm{out}}}\left( {\lambda ,\theta } \right)}}{{F\left( \lambda \right)}}}_{{\rm{Cavity}} - {\rm{Mode}}}$$

with a wavelength *λ*, viewing or emission angle *θ*, Planck’s constant *h*, speed of light in vacuum *c*, current *I*, elementary charge *e*, charge carrier balance factor *γ*, and EL spectrum for the embedded emitter *s*_EL_(*λ*), which is normally identified with the directly measurable photoluminescence (PL) spectrum *s*_PL_(*λ*)^29^. The second-to-last factor $\eta _{{\rm{rad}}}^ \ast \left( \lambda \right)$ is the effective radiative efficiency of the emitter, which is the ratio of the radiative and the total decay rate in the presence of a microcavity^10,11,29^. The out-coupled power spectrum per unit solid angle *P*_out_(*λ*,*θ*) is the only factor that contains an explicit angular dependency and is divided by the Purcell-Factor *F*(*λ*)^30^. Thus, the factor $\frac{{P_{{\rm{out}}}\left( {\lambda ,\theta } \right)}}{{F\left( \lambda \right)}}$ gives the emission affinity for a photon of a given wavelength *λ* under an angle *θ* within the microcavity. Accordingly, for a given emitter *s*_PL_(*λ*), only the last two factors in equation (1) define the final out-coupled spectra for the electroluminescent device. From an engineering point of view, it is the total thickness of the cavity, the reflectivity of the mirrors and the positioning of the emitting layers that define these two factors. Hence, they are referred to as the cavity mode. Influence of the microcavity on the final out-coupled spectrum for OLEDs has been widely analyzed before, with even preferential side emission observed previously^10,11,30,31^. However, none of these studies have investigated a possible sideward emission with respect to maximum efficiency.

Key influences on the cavity mode need to be considered to allow for effective beam shaping as demonstrated here. First, the thickness of the transparent contacts in the device plays a determining role. In analogy to a Fabry-Pérot resonator, a higher reflectance for the mirrors leads to a higher finesse, resulting in a narrower cavity mode and, consequently, a reduced full width at half maximum (FWHM) for the emission spectra^32^. Such arguments hold true for both ultra-thin wetting layer metal electrodes and metal oxides such as indium tin oxide, which are used in the device layout (cf. Fig. 1a)^15,33^.

Moreover, a change in the cavity length, meaning the resonance wavelength^34^, strongly influences the light emission from OLEDs^29,32,35^. In this study, the thickness of the device is adjusted through changes in the thickness of the highly conductive and transparent doped transport layers. Importantly, OLEDs are different from passive cavities because the active layer where EL originates, i.e., the emission layer (EML), accounts for only a small fraction of the total cavity thickness (typically, ~20 nm) and can be placed freely within the cavity. Thus, changes can be achieved for a given cavity length and variable EML position. For an unambiguous description, it is necessary to consider the distribution of the electromagnetic field in layered media^10,12,36^. OLEDs enclosing one field maximum are denoted as OLEDs of first order. Analogue OLEDs of second order show two maxima. The latter architecture allows for various ways of positioning the EML: in one of the two field maxima (optical maximum) or even in the minimum (optical minimum).

**Fig. 3:**
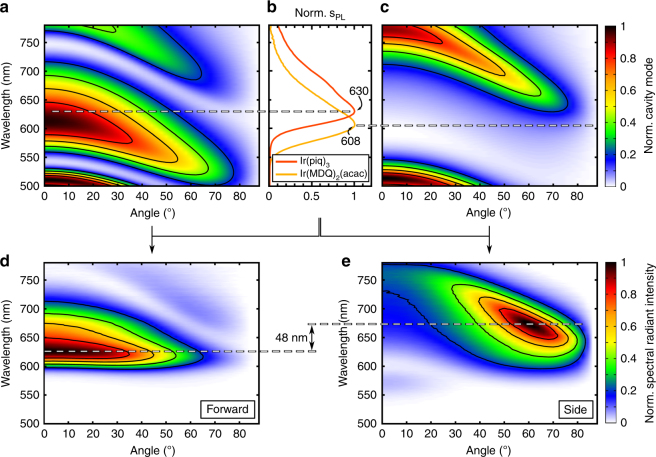
Simulation of the cavity mode and the SRI for beam-shaping OLEDs. Calculated cavity modes are shown for an OLED in the optical maximum (**a**) and the optical minimum (**c**), respectively; (**b**) Normalized PL spectra for the red emitters used. The peak intensities for both emitters are marked in the cavity modes to clarify the choice of emitter selected. Multiplying the cavity modes with the PL spectra explains the measured SRI as shown in **d** for forward emission and in **e** for sideward emission. The spectral difference between the maxima is 48 nm

Simulations demonstrate that OLEDs of higher order show narrower cavity modes, which bend to shorter wavelengths with increasing angles (compare Fig. 3a and Fig. 3c). The curvature follows a cosine-like behaviour, typical for a Fabry-Pérot resonator^34^. This effect is used for the desired angularly confined emission. Primarily forward emission (OLED 1 in Fig. 1) emerges from placing the EML close to the optical maximum. In our study, the cavity length is optimized for the dark red emitter Ir(piq)_3_. The resulting experimentally measured spectra (cf. Fig. 3d) show maximum intensity at 0° and 626 nm, dropping to half intensity at 48°. Outcoupling of another branch of the cavity mode is visible at higher angles but can be ignored due to its low intensity.

To realize a condition where the 0° emission is effectively suppressed (OLED 2 in Fig. 1), one has to place the EML in an optical minimum of the cavity (cf. Fig. S1, Supplementary Information). Accordingly, this results in a shift of the respective cavity mode (cf. Fig. 3c). As the SRI corresponds to the product of this mode and the PL-spectrum of the emitter used (Eq. (1)), it becomes clear that for emission at approximately 600 nm one can expect maximum emission at angles of 50–80°. Here, one challenge is the tail of the PL-spectrum towards long wavelengths (cf. Fig. 3b), because together with the increasing intensity of the cavity mode in the red wavelength regime, this results in a redshifted emission maximum, compared to the forward-emitting unit. To compensate for this effect, another emitter is chosen in the side-emitting unit: the red emitter Ir(MDQ)_2_(acac). Its PL-spectrum resembles that of Ir(piq)_3_ but is blueshifted by ~22 nm (Fig. 3b). This approach may seem counter-intuitive but represents one of the most important design steps towards colour stability between forward and sideward emission. The experimentally obtained SRIs are shown in Fig. 3d, e). Due to the aforementioned mode curvature, the resulting SRI for sideward emission peaks at higher viewing angles of approximately 60°. These SRIs for forward and sideward emission are compared to simulated values, which are calculated by multiplying the cavity modes with the respective PL spectra of the emitters, in Fig. S4 (Supplementary Information). The difference in wavelength between the experimental maxima in forward and sideward emission is 48 nm. This is slightly smaller than the value obtained from the spectra in Fig. 2c, which is because the latter refers to the maximum for the integrated radiant intensity. Without compensation through emitter choice, the peak differences would be as high as 66 nm, as simulated for the case of the emitter Ir(MDQ)_2_(acac). With a further blueshifted emitter molecule at hand, this colour difference can be further minimized.

**Fig. 4:**
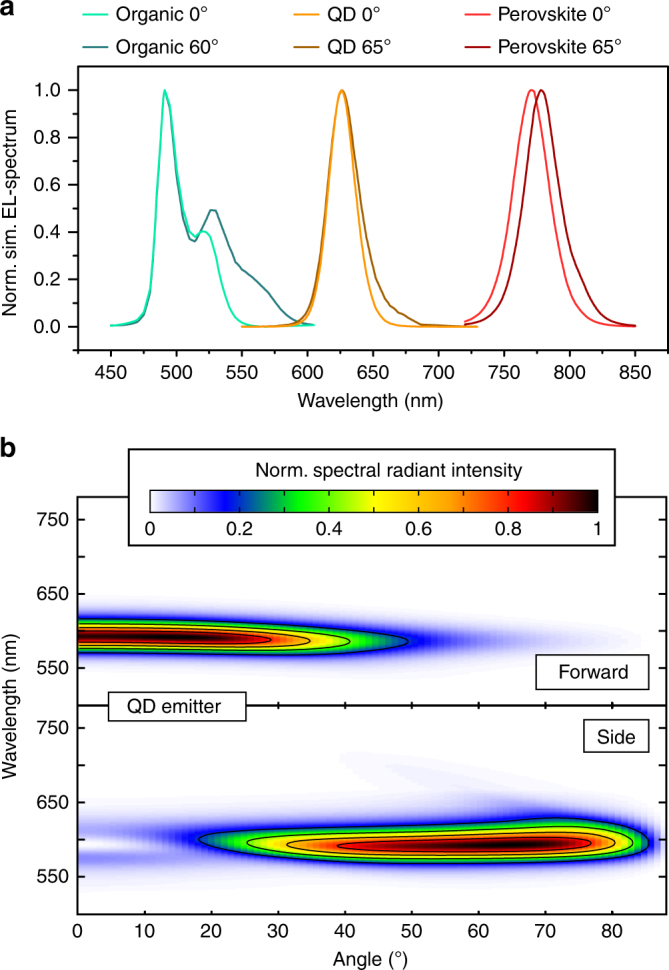
Increasing the colour stability using emitter materials with narrow PL spectra. **a** Simulated spectra for 0° emission (light lines) and for the angle of maximum side emission (dark lines) shown for three different emitting systems: organic, quantum dot, and perovskite; **b** Angular resolved SRI for the simulated QD-based emission in the forward and sideward configuration

Having applications in mind, often monochromatic emission is preferable. This refers to both the comparison of forward and side emission, as discussed above, but also to the chromatic stability of the two units themselves. Figure 3d shows a very colour-stable emission for the forward-emitting unit. However, the side emission follows the inherent bending of the cavity mode, which results in a colour shift with changing emission angle. To address this issue, emitting materials can be used that have narrow PL spectra. OLED emitters meeting this criterion are under investigation and their spectra can be found in the literature^37^. Additionally, our concept is easily transferable to other light sources based on layered stack architectures, providing emission spectra similar to those of light-emitting diodes based either on quantum dots (QDs)^38^ or on organometal halide perovskites^39^. Simulations were carried out using the respective PL spectra and adjusted thicknesses of the transport layers. The results for three model devices are shown in Fig. 4a. Light lines refer to the EL spectrum at 0° emission, and the dark lines refer to the angle of maximum intensity in side emission. For each emitter, the maxima for the spectra coincide quite well, and only for the organic emitter is a shoulder at approximately 530 nm more pronounced in side emission than in forward emission. Especially in the case of the QD emitter, the two spectra resemble each other very well, which results in very similar emission colour. Additionally, the colour stability as a function of the viewing angle for the QD system is excellent. As is shown in Fig. 4b, the shape of the spectrum is nearly conserved both in forward and in side emission. Hence, by incorporation of spectrally narrow emitters, beam-shaping architectures can be realized with very good colour stability.

**Fig. 5:**
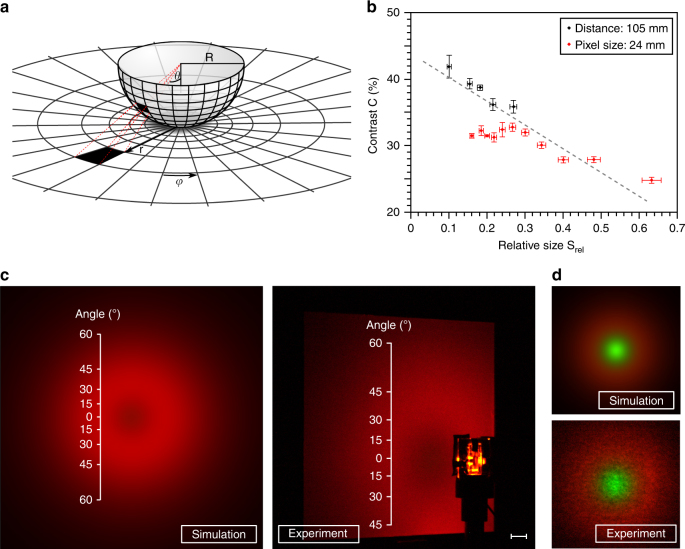
Observers position and contrast. **a** Scheme of the transformation needed to compare the measurements obtained on a spectro-goniometer and on a planar screen; **b** The contrast is shown with respect to the relative size S_rel_, which is the ratio of the size of the OLED to its distance from the screen. Black points were obtained by holding the distance constant; red points by holding the size constant. The smaller S_rel_, the better the contrast. The error bars represent the statistical confidence level of 1*σ*; **c** With the methods explained herein, it is possible to predict the beam shape from the spectro-goniometer data. Here the results for the red OLEDs developed in this contribution are shown. Scale bar is 10 mm; **d** To confirm that not only the brightness distribution but also the colours are calculated correctly, samples were fabricated that emit greenish light in the centre of the spot and red light towards the edges

Both the simulation and the measurement using a spectro-goniometer follow a radial symmetry with the light source in the centre of rotation, mimicking a spherical surface of observation. However, for many conceivable applications, this does not represent a very realistic model. Therefore, as introduced earlier, the analysis of the beam-shaping performance is carried out using a planar, diffusive screen (Fig. 1b). A subsequent image analysis enables one to obtain the brightness distribution. The results of spectro-goniometer and imaging-screen measurements are in direct correlation to each other via an azimuthal transformation, as sketched in Fig. 5a. The case where the projection source is in the centre of the sphere is called gnomonic projection. Assuming the OLED to be a point source and the aperture of the spectrometer as d*A* at a distance *R*, the corresponding area on the screen is d*A*′, which is located at a distance *r* from the north pole of the sphere (*θ*_north pole_ = 0°). Since the diameter of d*A* is much smaller than *R*, it can be interpreted as the surface element in spherical coordinates and the analogue d*A*′ in planar coordinates. Simple calculation yields for the irradiance *E*:

2 $$E_{{\rm{Goniometer}}} = \frac{1}{{{\rm{cos}}^3\theta }}E_{{\rm{Screen}}}$$

If the screen is at a distance *R*′ ≠ *R* from the OLED, equation (2) contains an additional factor $\left( {\frac{{R{\prime}}}{R}} \right)^2$. However, by normalizing the spectra, this factor cancels out again. It is worth mentioning that the surface element in spherical coordinates is not of constant size but depends on the azimuthal angle *θ*. However, the spectrometer comprises a constant aperture. This situation can be modelled by cylindrical coordinates. In this case, the transformation factor becomes $\frac{1}{{{\rm{cos}}^2\theta }}$. However, projecting the respective surface element on a two-dimensional plane does not give a filled surface. Hence, a true transformation should lie between those two models. This transformation highlights that the integrated radiant intensity for sideward emission has to be strongly enhanced (here a factor 2.7 to normal emission, cf. Fig. 1d). Otherwise, a hollow image, as shown in Fig. 1c for the sideward emission, cannot be realized.

Using the above transformation, the beam shape can be predicted if the spectral information is provided either by simulation or the goniometer measurement, which is of great importance for further optimization. Using CIE2006 colour matching functions and a subsequent linear transformation to RGB colour space, a displayable picture of the shape of the beam is achieved^40,41^. In Fig. 5c a calculated image is compared to a photograph. Both the brightness distribution and the colour are well reproduced. To prove the reliable reproduction of colours, OLEDs were built that emit green light in the centre of the spot and red light towards the sides (cf. Fig. 5d). The brightness-colour distribution is still well reproduced. Further information on this transformation is given in the Supplementary Information (cf. Fig. S5 and S6).

There remains an influence on the shape of the beam that has not yet been discussed. In the considerations above, the OLED has been treated as a point source. This gives rise to the question in which limits is this assumption right. There are two general ways to investigate this behaviour: one must vary either the size of the OLED or its distance to the screen. In the following discussion, the ratio of the size of the pixel *l*_dia_ and the distance *d*_scr_ will be analyzed, wherein the size denotes the length of the diagonal of the square pixel. This ratio will be referred to as the relative size $S_{{\rm{rel}}} = \frac{{l_{{\rm{dia}}}}}{{d_{{\rm{scr}}}}}$. For large values of *S*_rel_, rays emitted under various angles at different points of the source will be incident on the screen on the same spot, blurring the contrast. Experiments are carried out using beam-shaping OLEDs of *l*_dia_ = 28 mm pixel size. Using shadow-masks, the pixel size can be varied stepwise at a constant distance to the screen of 105 mm. As expected, the resulting contrast increases with decreasing relative size, as shown in black in Fig. 5b. More surprising is the result for a constant pixel size of *l*_dia_ = 24 mm. For relative sizes larger than 0.3, the values add nicely to the ones in black, but at approximately 0.3 the curve is kinked and starts to drop slightly again towards smaller *S*_rel_ values. Given the conservation of étendue in free space^42^, this cannot be a geometrical effect. Rather, it is based on greater noise in the spectra with increasing distance, due to longer exposure times of the camera. Assuming *I* is the maximum intensity, *i* is the intensity at the centre of the spot, ∆*I* is the contribution from constant noise, *C* is the contrast without noise, and $\tilde C$ is the contrast with noise, one obtains:

3 $$\tilde C = \frac{{\left( {I + {\mathrm{\Delta }}I} \right) - \left( {i + {\mathrm{\Delta }}I} \right)}}{{I + {\mathrm{\Delta }}I}} = \frac{{I - i}}{{I + {\mathrm{\Delta }}I}} \le \frac{{I - i}}{I} = C$$

Clearly, a non-zero constant noise for our camera system will limit the contrast values, as soon as the measured intensities become comparable to the noise floor. Increasing the driving current would counteract this effect, but to ensure that the interpretation of the data was as clean as possible, it was held constant over the whole measurement. These results also show that a definite prediction for the beam shape on the screen is only possible within the assumption of ideal measurement conditions.

So far, we have presented the possibilities offered by active beam shaping using planar, bare OLEDs. At the same time, this system offers functionality as a novel, adjustable platform in combination with additional secondary optics. As an example, we will discuss possible effects using primitive optical elements, in this case a half sphere and a prism, to demonstrate the variability of this promising concept.

**Fig. 6:**
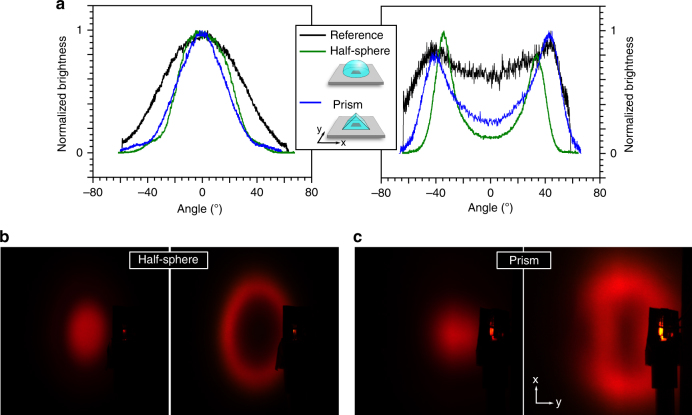
Adding secondary optical elements to active beam-shaping OLEDs. **a** The normalized brightness distribution along the evaluation line for forward (left) and sideward (right) emission is shown with and without additional optical elements placed on top of the OLED. The images on the projection screen obtained with a half sphere (**b**) and prism (**c**), respectively

The difference in refractive indices especially between the glass substrate and air leads to total internal reflections. By placing a glass half sphere on top of the substrate, one can suppress this effect and increase the device efficiency^43^. Additionally, the transition from glass to air at a planar interface results in a fanning out of emitted rays, which is efficiently reduced by the half sphere. Thus, a strong enhancement of the contrast is possible, as shown in Fig. 6. In comparison with the bare OLED (Fig. 1), the forward emission is more confined, and the side emission forms a sharp ring. The distance between the OLED and screen was kept constant during all measurements. Studying the brightness profile, the influence is obtained in a more quantitative way (Fig. 6a). In forward emission, the FWHM decreases to 43° compared to 62° for the reference. For sideward emission, the contrast between the centre of the spot and the maximum intensity increases from 26 to 85%. The corresponding spatial distance between the maxima is *D*_max_ = 82° and 67°.

Due to the physical shape of an OLED, no emission direction *φ* is preferred over others, leading to rotational symmetry for the beam, as shown in Fig. 1 and Fig. 6. However, some applications may require an asymmetric emission pattern. One way to break this symmetry is to add optical elements without circular symmetry, e.g., a prism, to the beam-shaping OLED. As illustrated in Fig. 6a, the prism covers the pixel completely. Due to its orientation, internal reflections in the *y* and *x* directions increase and decrease, respectively, compared to the reference. This behaviour leads to a strong change in the brightness distribution on the screen, as seen in Fig. 6c. Indeed, the beam shape now follows a mixture of the intrinsic radial symmetry of the OLED and the symmetry of the prism. The brightness in the forward emission and the x-direction gives a profile with a FWHM of only 40°. For sideward emission, one obtains a contrast value of 71% and *D*_max_ = 81° in the x-direction and a contrast of 60% at *D*_max_ = 46° in the y-direction.

## Conclusions

We have demonstrated that OLEDs are the first light sources that enable beam shaping without any further optical elements and a real-time continuous adjustment of the spatial brightness distribution. Several challenges were noted, such as the spectral drift of the emission colour, especially in the sideward emission, due to the curvature of the cavity mode. The use of emitters with spectrally narrower PL spectra would be an adequate response to that point. Furthermore, the emitted colour should be similar in both emission modes. This effect was addressed by utilizing two different emitters of similar spectral distribution but offset PL maxima.

Future work on this topic should focus on the development of green and blue beam-shaping OLEDs to cover the three primary colours. First tests have already been carried out and showed promising results. However, the influence of the *V*(*λ*) curve for human perception of colours^40^ works contrary to the curvature of the cavity mode, especially in the blue colour regime. On the one hand, this requires a far more elaborate sample design, but on the other hand the emission wavelength scales with the layer thickness, which means that process-based deviations in the thickness compared to the simulation have much more impact on the out-coupled spectra. These two facts complicate realization of the conceived optical design—the optical minimum in particular. However, given that these are experimental and not theoretical limitations, we are optimistic that further evaluation of this promising concept will be presented in future work, heading towards full colour active beam-shaping light sources.

Clearly, the ultra-thin, area light source character of OLEDs paired with the ability to control their angular emission properties solely through modulation of the driving signal opens a new opportunity to design and use complex, customized, and adjustable light sources and systems. The interplay between light sources and secondary optics warrants further investigation to make use of the full potential of this concept.

## Electronic supplementary material

Supplementary Information(PDF 837 kb)

Supplementary Material(GIF 5996 kb)
