# Supplementary material for: Real-time beam shaping without additional optical elements
Source: Light Sci Appl. 2018 Jun 20;7:18. doi: 10.1038/s41377-018-0014-0 (PMC6106982; doi:10.1038/s41377-018-0014-0)
Supplement: Supplementary file 1 — Supplementary Information(PDF 837 kb) [file 41377_2018_14_MOESM1_ESM.pdf]

Supplementary Information to:

Real-time beam-shaping without additional optical elements

Felix Fries*, Markus Fröbel, Pen Yiao Ang, Simone Lenk, Sebastian Reineke*

Dresden Integrated Center for Applied Physics and Photonic Materials (IAPP),

Technische Universität Dresden, Nöthnitzer Strasse 61, 01187 Dresden, Germany

*E-Mail: felix.fries@tu-dresden.de

sebastian.reineke@tu-dresden.de

1 Device Architecture

Fig. S1a) shows a detailed scheme of the device architecture. On top of the glass substrate, an ITO electrode is located. Then two pin-OLEDs are stacked on top, separated by a wetting layer electrode of only 10 nm metal. Doped layers are given with the corresponding doping ratio. In the case of BPhen:Cs the doping ratio is chosen to achieve a conductivity of 10^{-5} S/cm. Additionally, the electrical connections are indicated in Fig. S1a).

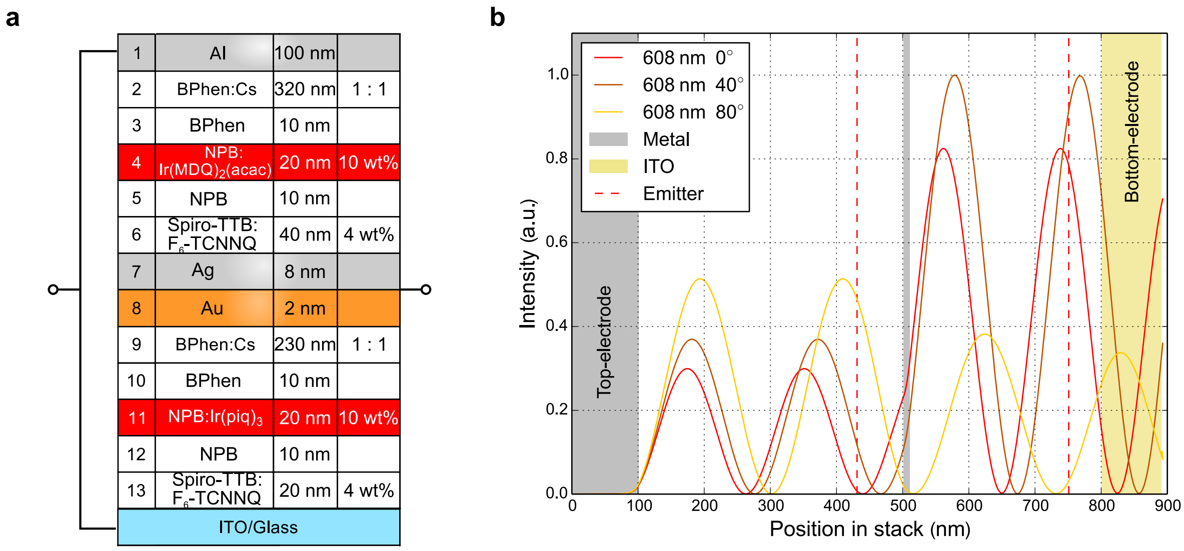

Figure S1 Stack Architecture Detailed scheme of the device architecture. Long names of the materials can be found in the materials section of the main article.

Solving Maxwell's equations for the stratified device, one obtains a distribution of the intensity of the electric field.[1,2] In the simulation used in this contribution the field is, of course, taken into account. The visualization of the location of the emissive layer within this field helps to interpret the out-coupled spectra. As shown in Fig. S1b), the emission layer for side emission is located close to a field minimum at 0°. By increasing the viewing angle, a local maximum is shifted to the position of the emission layer. Note that for simplicity here the emission zone is assumed to be infinitesimal thin. The emission zone in the forward emission unit faces a local maximum at 0°, which increases in intensity with increasing viewing angle up to 35° and only then starts to drop.

2 PWM

Using PWM, the ratio of primarily side emission to primarily forward emission can be adjusted. In the main article three examples show how the image of the beam shape can be modified (Fig 1). Of course, this can also be measured with the spectro-goniometer. In Fig. S2, the angle-dependent spectral radiant intensity (SRI) is presented for different waveforms. From the top-left to the bottom-left the ratio of forward to side emission changes from 100% duty cycle to 0% duty cycle in 20% steps. All the spectra are individually normalized to their maximum value at 0°. Integrating each of the spectra in Fig. S2 over the wavelength, one obtains the irradiance development shown in Fig. S3. The duty cycle increment here is 10%.

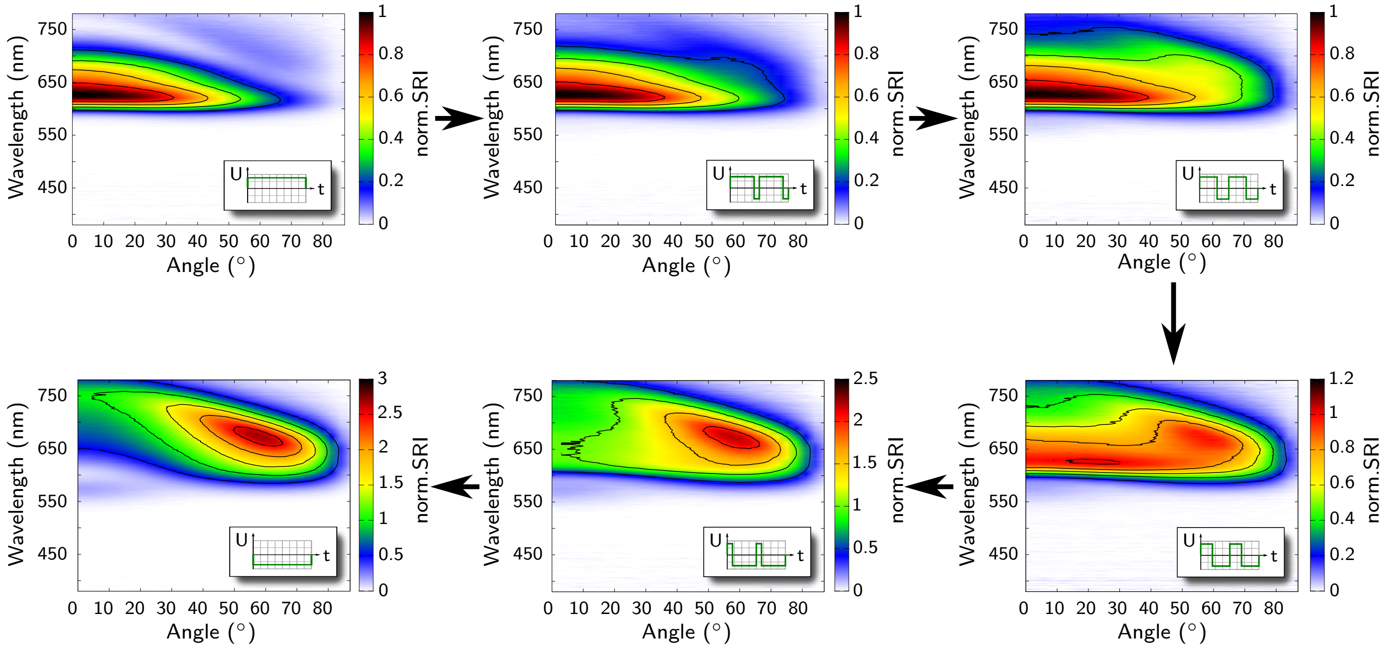

Figure S2 Spectra under AC conditions Changing the duty cycle, the spectra continuously change from forward to side emission. Duty cycle increment is 20%.

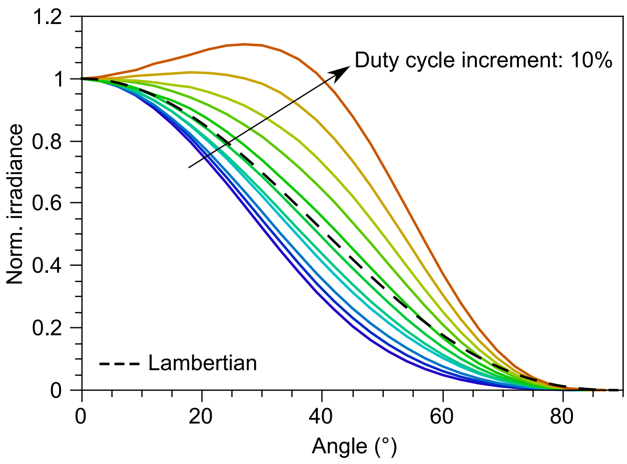

Figure S3 Irradiance under AC conditions. Integrating the spectra for different PWM signals over the wavelength gives the irradiance development from forward to side emission for various duty cycles. For comparison the profile of a Lambertian emitter is shown.

3 Comparison of simulated and experimental spectra

As shown in Fig. S4, the simulated spectra predict qualitatively quite well the experimentally measured behaviour. For side emission, low intensity can be seen at 0°, rising to a maximum around 60°. The forward-emission unit shows strong emission around 608 nm and 0°, dropping in intensity fast with increasing viewing angle. Also a parasitic mode around 700 nm and 65° is reproduced, even though in the experiment it is much less pronounced.

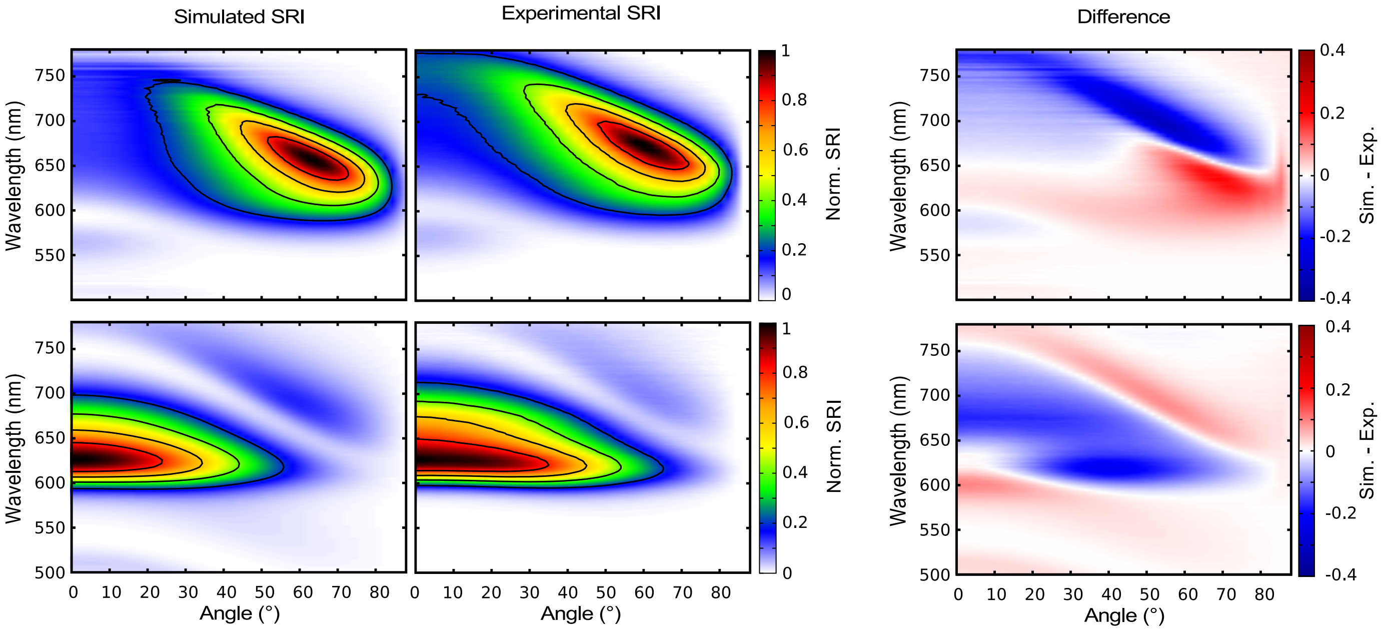

Figure S4 Simulated and measured spectra. The left column shows the simulated spectra of the side-emission unit and the forward-emission unit, respectively. Beside it, in the middle column, the experimentally measured spectra are shown for comparison. The qualitative behaviour is reproduced fairly well. For quantitative analysis, difference spectra were calculated, as shown in the right column.

For a quantitative analysis, the difference between the two spectra (simulation minus experiment) is calculated and shown in the right column. In side emission it becomes clear that the experimental spectra are slightly red shifted compared to the simulation, which hints that the cavity length and thus the resonance wavelength of the resonator is not exactly as expected. In forward emission, differences in the shape are visible that way. The simulation drops faster than the experimental data and again the parasitic mode is more pronounced.

Adjusting the layer thickness of several layers in the simulation may lead to a better agreement and thus could provide further information on the processing procedure.

4 Gnomonic Transformation

As written in the main text, the transition between the spectro-goniometer and the planar screen is described by an azimuthal transformation, precisely a gnomonic projection. Treating the OLED as a point source, the emitted light symmetric with respect to the azimuthal angle φ, the detector at a distance R, and its aperture $\mathrm{d}A$ being much smaller then R, which than is equal to the surface element in spherical coordinates:

$$\mathrm{d}A = R^2 \sin\theta \mathrm{d}\varphi \mathrm{d}\theta \quad . \tag{1}$$

Using the same notation as in the main text, the respective area on the flat screen, can be written as the surface element in polar coordinates $\mathrm{d}A'$, which is at a distance r from the sphere's north pole:

$$\mathrm{d}A' = r\mathrm{d}r\mathrm{d}\varphi \quad , \tag{2}$$

where the relation between r and R is given as:

$$r = R \cdot \tan\theta \quad . \tag{3}$$

With this the transformation between the two measurement systems is straightforwardly obtained:

$$\mathrm{d}r = \frac{\partial r}{\partial R}\mathrm{d}R + \frac{\partial r}{\partial \theta}\mathrm{d}\theta \overset{\mathrm{d}R=0}{=} \frac{R}{\cos^2\theta}\mathrm{d}\theta \quad ,$$

$$\Rightarrow \mathrm{d}A' = \frac{1}{\cos^3\theta}\mathrm{d}A \quad . \tag{4}$$

With the definition of the irradiance E as the derivative of the photonflux $d\Phi$ with respect to the detecting surface dA, one gets:

$$\Rightarrow E(\lambda)_{\text{Goniometer}} = \frac{1}{\cos^3\theta} \cdot E(\lambda)_{\text{Screen}} \quad . \tag{5}$$

As the aperture of the detector is constant, assuming a cylindrical symmetry, rather than a spherical, appears to be appropriate. This results in:

$$E(\lambda)_{\text{Goniometer}} = \frac{1}{\cos^2\theta} \cdot E(\lambda)_{\text{Screen}} \quad . \tag{6}$$

In Fig. S5, the measured brightness distribution on the screen (black) is compared to the integrated irradiance, obtained using the spectro-goniometer (non-filled red dots). Especially in the side emission, a strong difference in the shape is obvious. The contrast between 0°-emission and the maximum is way higher in the case of the goniometer. Furthermore, the profile peaks around 60° compared to 40° on the screen. When multiplying the goniometer profile with $\cos^2(\theta)$, those data resemble the black line very well. Both the contrast and the maximum angle is reproduced. In forward emission direction, the drop of intensity coincides, as well. Actually, it is surprising that the cosine of the angle to the power of two gives the best results, as a power between two and three was expected to model the transformation most realistically. Obviously, some of the

assumptions made are not completely fulfilled. For example, in Fig. 4b) it can be seen, that the sample may not be treated as a point source. Nevertheless, accepting this factor two, the profiles of the measurements are highly comparable.

With this information the expected beam shape on a screen can be calculated from the spectro-goniometer data or even from simulation data, which opens further possibilities for sample optimization. The first step to be done is to transform the spectra. In Fig. S6, this is shown for the experimental data. Of course, this transformation leads to an enhancement of low-angle parts, shifting the maximum to a lower angle. Subsequently, the CIE-XYZ coordinates are calculated for each angle, using the CIE2006 colour-matching functions.[3]

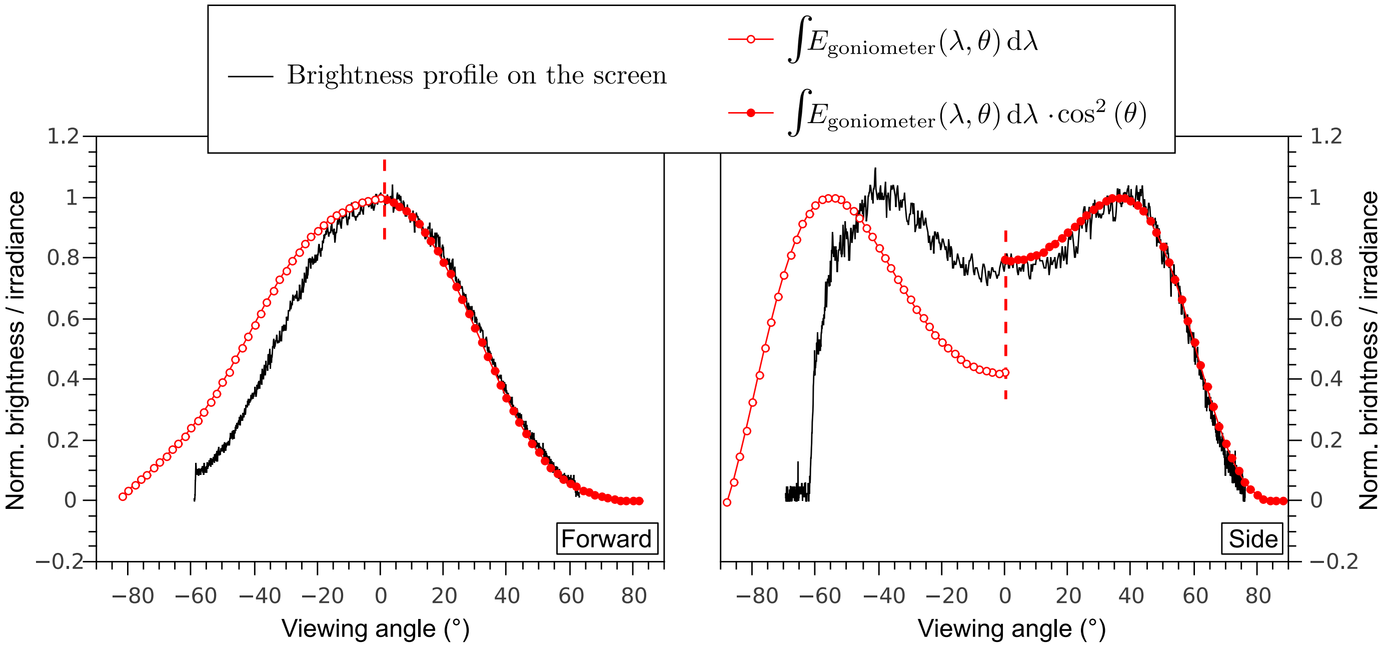

Figure S5 Brightness profiles under different conditions. The black lines show the brightness distribution on the screen. The abrupt drop around -60° in side emission is caused by the shadow of the sample holder. Red lines with white dots show the irradiance measured at the spectro-goniometer. Applying a transformation factor $\cos^2(\theta)$ on the latter, both measurements coincide as shown in red lines with solid dots.

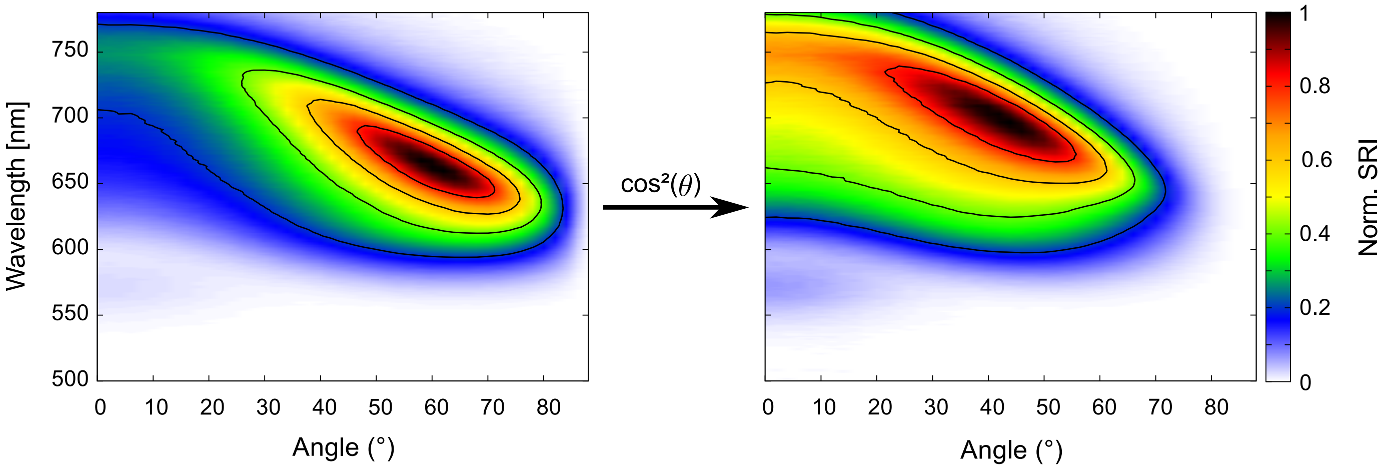

Figure S6 Spectral transformation. Applying the azimuthal transformation on the spectral radiant intensity, the contrast between 0° and maximum emission gets reduced.

The last step towards a true colour image is a linear transformation ($\mathcal{M}_{3\times3}$) from the CIE-XYZ to a displayable RGB colour space. In case of the sRGB space with D65 as reference white point this reads:[4]

$$\begin{pmatrix} R \\ G \\ B \end{pmatrix} = \begin{pmatrix} 3.24 & -1.54 & -0.50 \\ -0.97 & 1.88 & 0.04 \\ 0.06 & -0.20 & 1.06 \end{pmatrix} \begin{pmatrix} X \\ Y \\ Z \end{pmatrix} \quad (7)$$

This gives an 1D-array containing one RGB-tuple for each angle, which has to be multiplied with the calculated integrated irradiance distribution (equation (6)). Note that with this, the ratio between the R,G, and B coordinates is unchanged which results in a sole change of the brightness, but not of the respective colour. Assuming again azimuthal symmetry this 1D-colour-brightness distribution can be expanded to a 2D-colour-image.

References

[1] Sipe JE. New Green-function formalism for surface optics *J Opt Soc Am B* 1987; **4**: 481-489.

[2] Heavens OS. *Optical Properties of Thin Solid Films* Dover Publications: New York, 1991

[3] Commission Internationale de l'Éclairage. Fundamental chromaticity diagram with physiological axes–Part I. Technical Report, CIE 170-1:2006, 2006.

[4] Hunt RWG. *The reproduction of colour* 6th edn. Wiley: Chichester, 2004
