# Supplementary figures and images for: Real-time beam shaping without additional optical elements
Source: Light Sci Appl. 2018 Jun 20;7:18. doi: 10.1038/s41377-018-0014-0 (PMC6106982; doi:10.1038/s41377-018-0014-0)

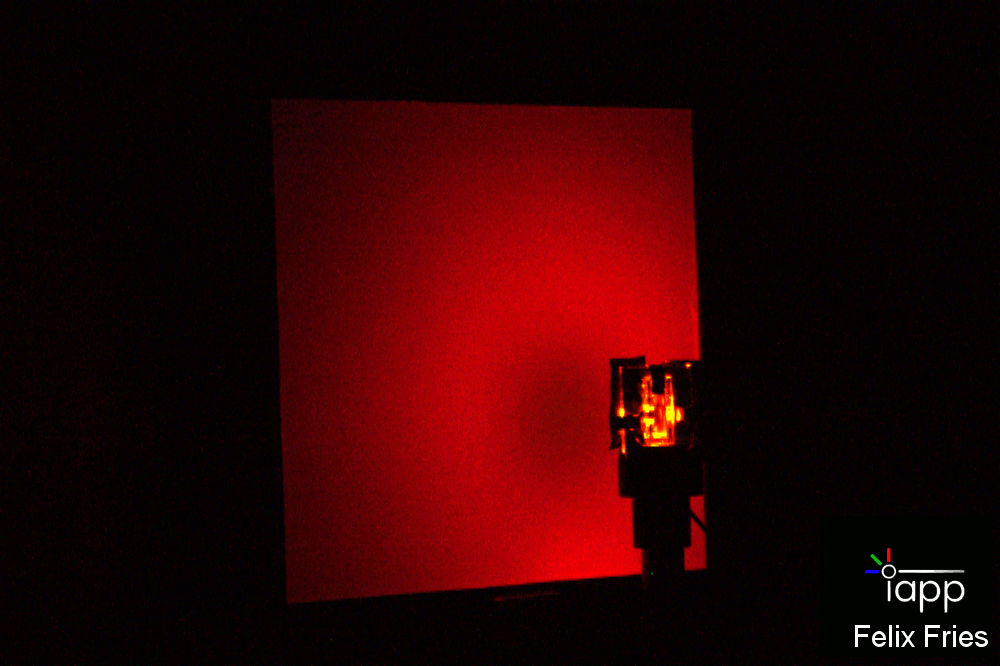

Supplement: Supplementary file 2 — Supplementary Material(GIF 5996 kb) [file 41377_2018_14_MOESM2_ESM.gif]
